# Detecting change-points in preclinical rheumatoid arthritis biomarkers using Bayesian multivariate segmented regression

**DOI:** 10.64898/2026.05.22.26353892

**Published:** 2026-05-25

**Authors:** Yonatan F. Wolde, Alexandria M. Jensen, Brandie D. Wagner, Jess D. Edison, Marie L. Feser, Michael Mahler, Kevin D. Deane, Kevin P. Josey

**Affiliations:** 1 Department of Biostatistics and Informatics, Colorado School of Public Health; 2 Quantitative Sciences Unit, Stanford School of Medicine; 3 Department of Medicine, Uniformed Services University of the Health Sciences School of Medicine; 4 Division of Rheumatology, University of Colorado Anschutz Medical Campus, Aurora, Colorado, USA; 5 Werfen, San Diego, California, USA

**Keywords:** rheumatoid arthritis, autoantibodies, change-point, Bayesian inference, segmented regression, biomarkers, ACPA, rheumatoid factor

## Abstract

**Background:**

Rheumatoid arthritis (RA) has a preclinical period characterised by elevations in serum autoantibodies. Identifying the timing and magnitude of autoantibody trajectory changes may inform screening strategies and preventative interventions.

**Methods:**

Using a Bayesian multivariate segmented regression, we jointly modelled longitudinal autoantibody trajectories from two Department of Defense Serum Repository cohorts (Sample A: 209 matched case–control pairs, 1566 samples, six biomarkers; Sample B: 309 cases with two matched controls each, 2758 samples, eight biomarkers). Change-points and magnitudes of change were estimated simultaneously under a multivariate likelihood with an unstructured residual correlation matrix.

**Results:**

In Sample A, five of six biomarkers exhibited pre-diagnostic trajectory shifts with 95% highest posterior density intervals excluding zero. RF-IgM demonstrated the earliest change-point at 8.10 years before diagnosis (95% HPDI: −10.47, −5.73), followed by ACPA-IgG at 7.43 years (95% HPDI: −9.33, −5.76). In Sample B, only the four IgG isotypes showed pre-diagnostic shifts, with anti-CCP3 (IgG) earliest at 7.00 years (95% HPDI: −8.48, −5.29). A composite metric integrating timing and magnitude reordered rankings.

**Conclusions:**

This Bayesian framework enables simultaneous estimation of change-points and magnitudes across correlated autoantibodies while fully characterising uncertainty, offering a complementary approach to prior divergence-based methods for understanding preclinical RA autoimmunity.

## Introduction

Rheumatoid arthritis (RA) is an autoimmune disorder that affects 0.5-1% of the population.^1,2^ The disease imposes considerable burden through systemic inflammation, joint damage as well as increased cardiovascular mortality, making early identification and intervention an important public health priority. Recent findings in RA pathogenesis have revealed a well-characterised preclinical stage that precedes clinical arthritis by years or even decades, presenting opportunities for early disease detection and prevention strategies.

This preclinical stage is characterised by progressive development of autoimmune responses that can be detected by the emergence and evolution of serum autoantibodies.^3-7^ Multiple autoantibodies have been identified in preclinical RA, including biomarkers that are well-established in RA such as rheumatoid factor (RF) and anti-citrullinated protein antibodies (ACPA). Notably, ACPA is a general class of autoantibodies, and the most common version which is used in clinical care is the anti-cyclic citrullinated peptide (anti-CCP) antibody. Both RF and ACPA can be of a variety of isotypes including immunoglobulin (Ig) A, IgM and IgG. In addition, there are emerging data showing that additional autoantibodies, including antibodies to specific citrullinated proteins as well as to isoenzymes of peptidyl arginine deiminase (anti-PAD) can be present in preclinical RA.^8-10^

Evaluating the temporal dynamics of these autoantibodies during the preclinical stage serves multiple purposes. From a biological perspective, characterising the sequence and timing of autoantibody emergence informs the progressive changes in immune tolerance and the body’s subsequent autoimmune responses. From a clinical perspective, identifying which autoantibodies appear earliest, and demonstrating the most pronounced changes, helps researchers develop biomarker-based screening strategies to identify at-risk populations. Regarding the latter point, understanding these temporal patterns may inform the prediction of the timing of future onset of RA, as well as the potential timing of preventive interventions, which are areas of intense focus given recent studies demonstrating the potential for delaying or preventing RA onset in high-risk individuals.^11,12^

Previous investigations into preclinical RA autoantibody trajectories have provided valuable insights but with methodological limitations. In particular, using a longitudinal dataset derived from the Department of Defense Serum Repository (DoDSR), Kelmenson and colleagues^13^ modelled six autoantibodies in preclinical RA using mixed-effects cubic B-splines. These analyses identified an approximate change-point of each autoantibody by selecting the earliest time at which the cubic function of cases and controls began to diverge. However, while prior studies, such as Kelmenson and colleagues,^13^ identified differences between cases and controls, they did not jointly model correlated autoantibodies, fully characterise the uncertainty of the change-point, or formally incorporate the magnitude of autoantibody elevation. Our study addresses these specific methodological gaps by introducing a Bayesian framework designed to simultaneously estimate change-points and their associated magnitudes across multiple, correlated autoantibody trajectories. Furthermore, there have since been additional autoantibody biomarkers measured within this rich DoDSR-derived dataset that can be assessed.

Building on this methodological foundation, we investigate when each autoantibody biomarker changes trajectory in the preclinical stage of RA development to determine which biomarkers show the earliest and most pronounced changes in serum concentrations. Our approach jointly models multiple biomarkers to account for their interdependencies, offers a full characterisation of the uncertainty through Bayesian posterior distributions, and incorporates both the timing and magnitude of change in the biomarker’s serum concentration. This framework is applied to two complementary autoantibody biomarker panels. The first of these panels contains six autoantibodies previously studied in this DoDSR cohort, enabling direct comparison with prior findings. The second panel is a set of eight novel autoantibody markers measured using advanced multiplex technology, expanding our understanding of the preclinical autoimmune response.

## Methods

### Data Collection and Study Design

We used a cohort of individuals who developed clinical RA and matched controls that was obtained from the DoDSR.^8,13,14^ In brief, this cohort includes longitudinal clinical data and serum samples obtained prior to and after a diagnosis of clinical RA based on established classification criteria or diagnosis by a board-certified rheumatologist from individuals who developed RA while on active-duty military service. Use of the anonymised clinical data and biospecimens has been approved by Institutional and Regulatory Review Boards at the University of Colorado, Walter Reed National Military Medical Center and the U.S. Army Medical Research and Development Command, Office of Research Protections (ORP), Human Research Protection Office (HRPO).

We evaluated two subsets of subjects from this larger cohort: Sample A, which included cases matched to a single control with prior biomarker testing; and Sample B, which included cases that have two matched controls. Controls were matched to cases based on sex, race, age, and region of enlistment, and were selected to have a similar longitudinal timespan to that of their matched case. In each subset, only observations with complete biomarker data were included. A summary of the final datasets is described in Table 1.

In Sample A, which is the same set used in the publication by Kelmenson and colleagues,^13^ we evaluated six biomarkers: ACPA IgA, IgG, and IgM, and RF IgG, IgA, and IgM. These assays were performed on an enzyme-linked immunosorbent assay (ELISA; Inova Diagnostics, Inc., San Diego, California). In Sample B, we evaluated eight autoantibody biomarkers that were assessed using a novel testing platform that assessed autoantibody biomarkers including both IgG and IgA isotypes of antibodies to cyclic citrullinated peptide-3 (anti-CCP3, the target of which contains a proprietary mix of citrullinated antigens), and antibodies to the following specific citrullinated proteins: citrullinated vimentin-2 (anti-citVim2), cit-fibrinogen (anti-citFib) and citrullinated histone-1 (anti-citHis1). These eight biomarkers were evaluated on a novel, fully automated particle-based multianalyte technology (Inova Diagnostics, Inc.) and the Aptiva system, which uses paramagnetic particles with unique signatures and a digital interpretation system, as described previously.^15^ These eight biomarkers were selected because, in prior analyses using this same cohort and using dichotomous cut-offs for each marker, their IgG versions were found to be positive at meaningfully higher rates in RA cases compared to controls.^8^

### Statistical Analysis

We employed a Bayesian segmented linear mixed modelling approach to jointly analyse serum biomarker trajectories over time.^16-18^ We applied this approach to Sample A and Sample B as independent analyses; the two cohorts were not pooled. The primary response for each observation was a vector of raw serum biomarker measurements which we assume follow a truncated multivariate normal distribution, with each observation bounded by the assay’s lower and upper detection limits. For participant i=1,2,…,n, measurement within a participant $j=1,2,\ldots ,m_{i}$, l=1,2,…,p, denoting case-control pairs (we use l[i] to identify patient pairs corresponding with participant *i*), and for biomarker k=1,2,…,K (*K* = 6 for Sample A; *K* = 8 for Sample B), the multivariate outcomes are denoted with $Y_{ij}={\left[Y_{ij1},Y_{ij2},\ldots ,Y_{ijK}\right]}^{T}$ with each element $Y_{ijk}$ constrained to lie between a lower bound of 0 (i.e., $L={\left[L_{1},L_{2},\ldots ,L_{k}\right]}^{T}=0_{K}$) and an upper bound specific to the *k*^th^ biomarker’s maximum detectable value, denoted as $U={\left[U_{1},U_{2},\ldots ,U_{K}\right]}^{T}$. The response distribution is

$$Y_{ij}∼\text{Truncated Norml}[\mu (X_{i},T_{ij};\alpha_{l[i]},\beta_{1},\beta_{2},\gamma ,\delta ),\Sigma ,L,U]$$

where Σ is the variance-covariance matrix with unstructured correlation among biomarkers.^19-21^ The mean structure is defined as:

$$\mu (X_{i},T_{ij};\alpha_{l[i]},\beta_{1},\beta_{2},\gamma ,\delta )=\alpha_{l[i]}+\beta_{1}X_{i}+\beta_{2}T_{ij}+\gamma (W_{ij}\times X_{i})$$

where *X_i_* ∈ {0,1} is an indicator for whether participant is diagnosed with RA, $T_{ij}$ is the time before diagnosis of the case in the case-control pair, and $W_{ij}=\mathrm{pmax}\{0,T_{ij}-\delta \}$ with $\delta ={\left(\delta_{1},\delta_{2},\ldots ,\delta_{K}\right)}^{T}$ representing the biomarker specific changepoints. The paired random intercept $\alpha_{l}∼N(\theta ,\Omega )$, where $\Omega =\text{diag}(\omega^{2})$ is the diagonal matrix consisting of entries $\omega ={\left(\omega_{1},\omega_{2},\ldots ,\omega_{K}\right)}^{T}$, accounts for the correlation induced by repeated measurements on the same participant^19,20^ and captures variation due to time-invariant characteristics (e.g., sex, race, and age at diagnosis). γ quantifies the magnitude change (change in slope) among RA cases. Furthermore, Σ is parametrised as $\Sigma =\text{diag}(\sigma^{2})⊙\Phi$ where Φ is a (*K* × *K*) correlation matrix and $\text{diag}(\sigma^{2})$ is the diagonal matrix of measurement variances with $\sigma ={\left(\sigma_{1},\sigma_{2},\ldots ,\sigma_{K}\right)}^{T}$. Non-informative priors were placed on all regression parameters and hyperparameters^22,23^; each change-point received a uniform prior over [−20, 10] years and the correlation matrix Φ received a Lewandowski-Kurowicka-Joe (LKJ) prior. Additional prior specifications are given in the Supplement.

In addition to the change-points $\delta_{k}$ and post-change slopes $\gamma_{k}$, we also summarise both jointly through a composite time $\tau_{k}$, defined as the earliest time satisfying $\text{Pr}\{\gamma_{k}(\tau_{k}-\delta_{k})>0\;|\;X_{i}=1\}=0.9$. This is the point at which there is high posterior probability that the biomarker’s mean among cases has departed from its baseline trend. Biomarkers in Table 2 are ranked by $\tau_{k}$. When this posterior probability does not reach 0.9 anywhere within the search window, the composite time is reported as NA. A sensitivity analysis treating the upper detection limit as right-censored rather than truncated, with the residual covariance restricted to be diagonal because of computational constraints in Stan, is reported in the Supplement.

Posterior inference was conducted using Markov Chain Monte Carlo (MCMC) sampling in Stan.^24^ Five chains, each containing 20 000 iterations, were simulated, with the first 10 000 discarded as a warm-up and a thinning interval of ten applied. The prior distributions and MCMC parameters were selected to be non-informative and to produce posterior samples with good mixing, as assessed by diagnostic plots. Inference on the change-point and magnitude of change was based on 95% high posterior density credible intervals.^25^ R (v 4.5.0) and Stan statistical computing software were used to implement the analysis. For further details on the code used in our analysis, refer to the GitHub repository https://github.com/yonwol3/ra-biomarker

## Results

Table 1 summarises demographic characteristics of participants with and without RA. Sex, race, and age at diagnosis were balanced between RA and non-RA groups. Among individuals who developed RA, the median age at diagnosis was 36.2 years for Sample A and 34.7 years for Sample B.^26^

**Figure 1.**
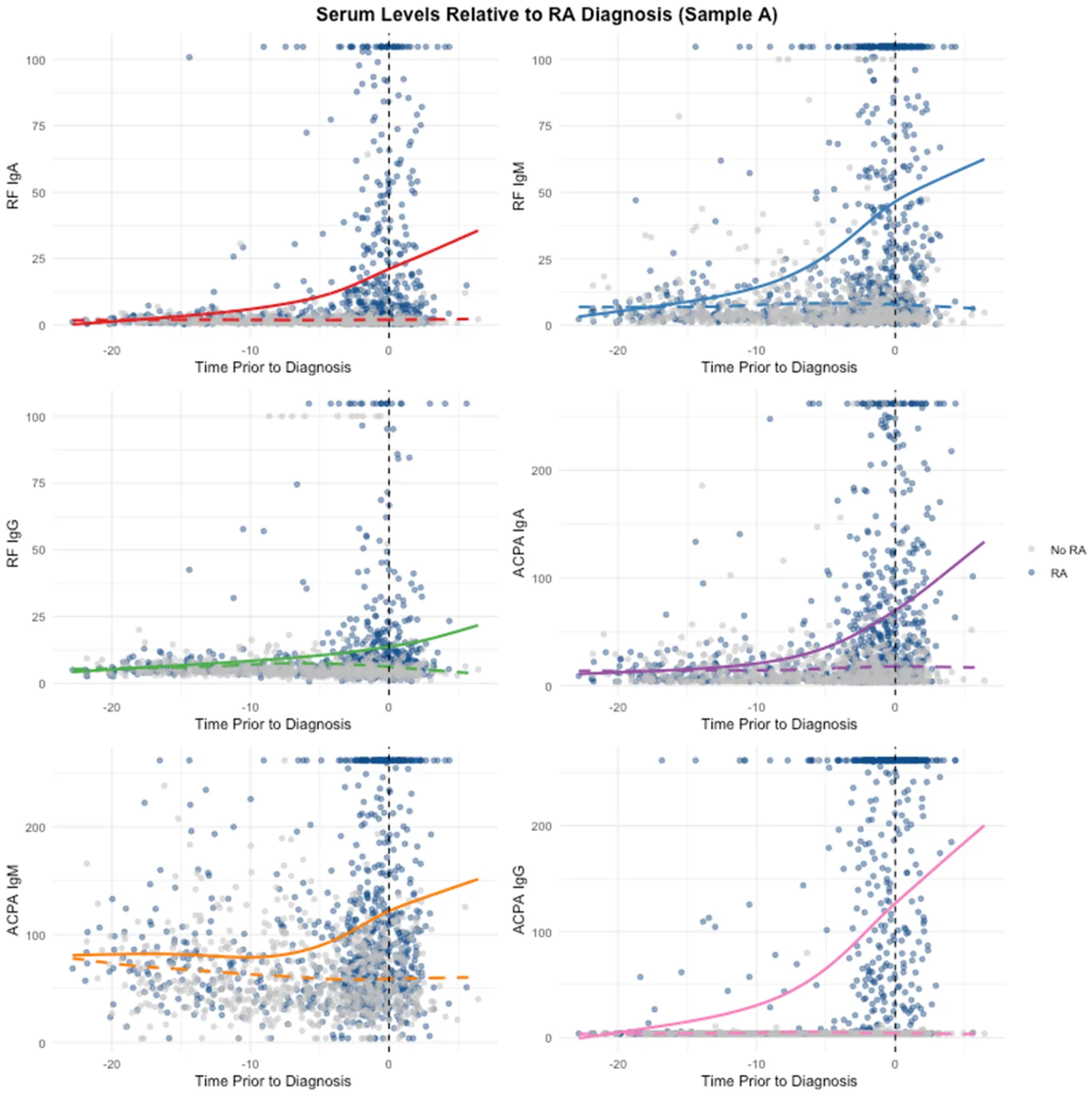
Scatterplots of the serum antibody levels for six biomarkers (RF-IgA, RF-IgM, RF-IgG, ACPA-IgA, ACPA-IgM, and ACPA-IgG) plotted against time (in years) before rheumatoid arthritis (RA) diagnosis. Trends for cases (solid curves) and controls (dashed curves) are shown. Time = 0 (vertical dashed line) indicates the time of diagnosis of RA in the cases. Each plot is overlaid with a smoothing spline (6 degrees of freedom) to illustrate temporal trends in biomarker levels. Note the apparent rise in concentration for five of the six biomarkers in cases (solid lines) prior to the time of diagnosis (t=0), except for RF-IgG.

**Figure 2.**
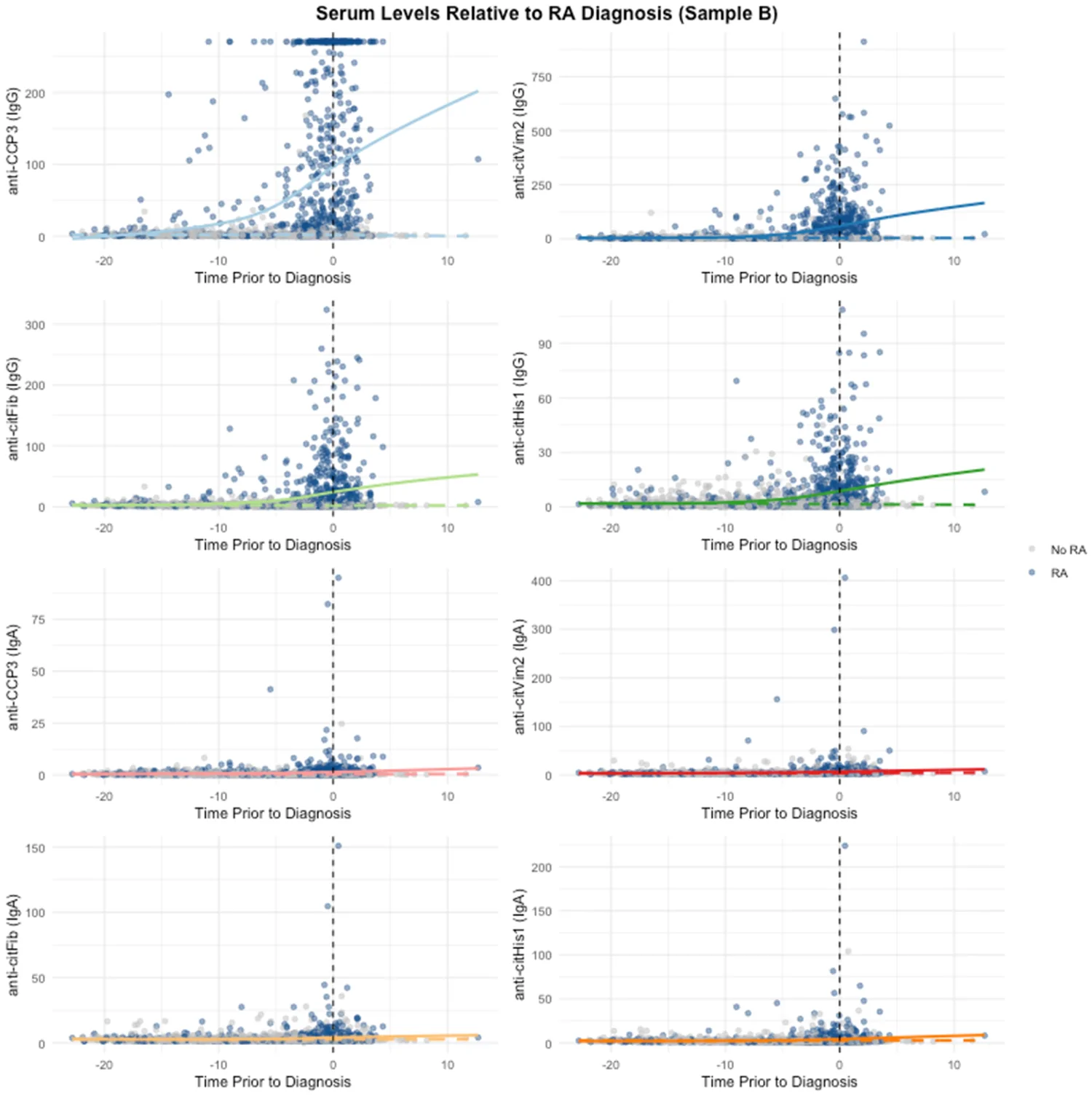
Scatterplots of the serum antibody levels for eight biomarkers (both IgG and IgA variants of anti-CCP3, anti-citHis1, anti-citVim2, and anti-citFib) plotted against time (in years) before rheumatoid arthritis (RA) diagnosis. Trends for cases (solid curves) and controls (dashed curves) are shown. Time = 0 (vertical dashed line) indicates the time of diagnosis of RA in the cases. Each plot is overlaid with a smoothing spline (6 degrees of freedom) to illustrate temporal trends in biomarker levels. Observe that discernible pre-diagnostic increases in concentration are primarily visible among the IgG isotypes for cases (solid lines).

Figures 1 and 2 provide a visual description of the temporal trends in serum biomarker levels among cases and controls. Figure 1 displays scatterplots with smoothed averages for six biomarkers tested in Sample A, showing that five markers increase before diagnosis. The exception is RF-IgG, which does not show a pre-diagnostic rise in concentration; instead, a change in its trajectory appears to occur after diagnosis. Similarly, Figure 2 illustrates scatterplots for the eight biomarkers tested in Sample B, where some of the biomarkers exhibit discernible change before diagnosis among RA cases. For the IgA biomarkers, such as anti-CCP3(IgA), anti-citHis1(IgA), anti-citFib(IgA), and anti-citVim2(IgA), the change before diagnosis is indiscernible. In both figures, the post–change trends are approximately linear even though the smoothing spline could accommodate more complex nonlinear trends, supporting our proposed segmented regression model with unknown change-points.

**Figure 3:**
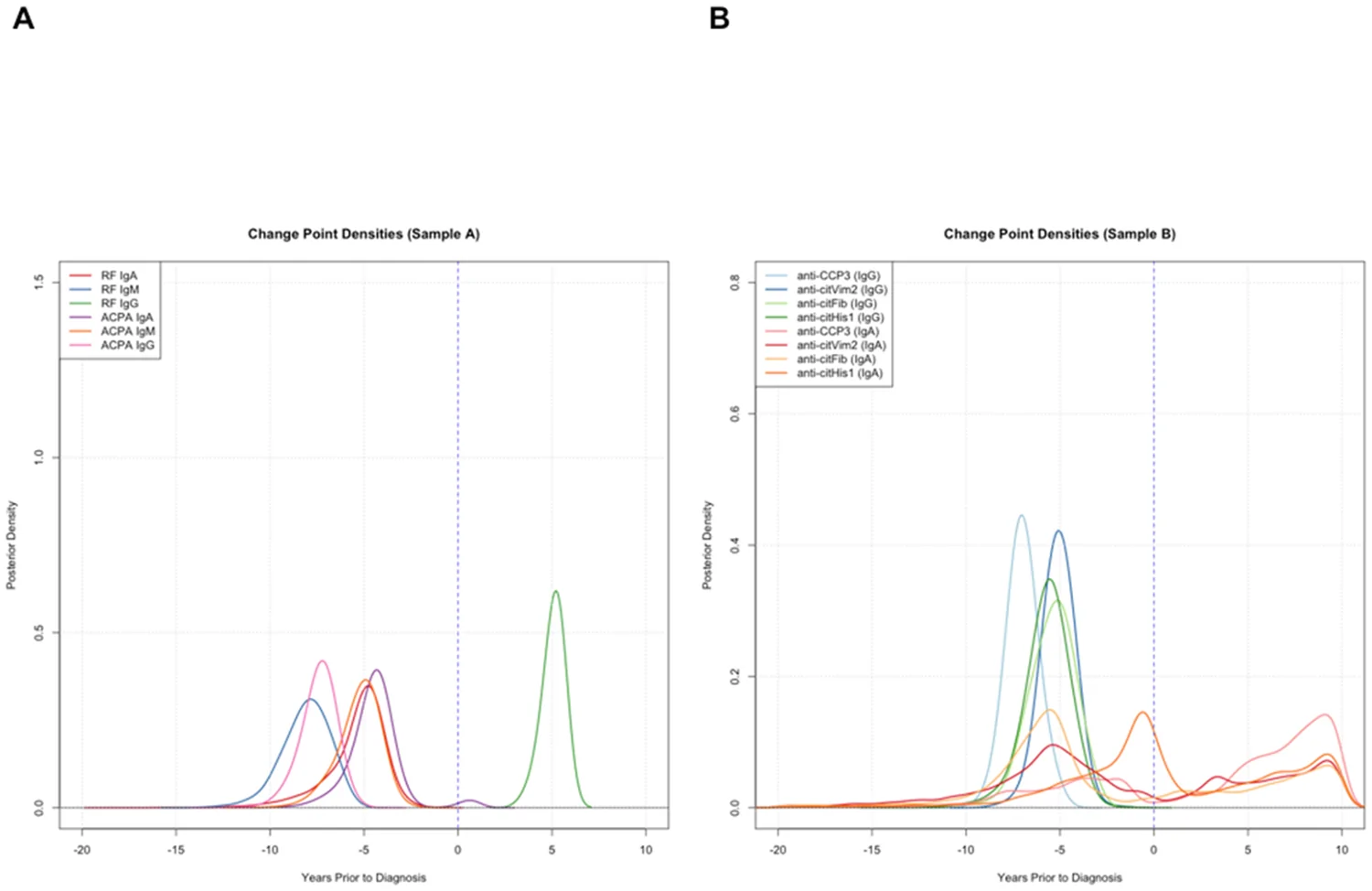
Posterior probability density functions of the change-point parameter for each biomarker. The x-axis represents years relative to RA diagnosis, with negative values indicating the pre-diagnostic period. Peaks to the left of zero indicate a high posterior probability of a pre-diagnostic change-point. **Left Panel A** depicts posterior change-point density for the six biomarkers in Sample A (labels in the top-left corner), while **Right Panel B** depicts posterior change-point density for the eight biomarkers in Sample B (labels in the top-left corner). The peak of each density plot corresponds to the most probable change-point estimate. RF=rheumatoid factor; Ig=immunoglobulin; ACPA=anti-citrullinated protein antibody; CCP3=cyclic citrullinated peptide-3; cit=citrullinated; His=histone; Vim=vimentin; Fib=fibrinogen.

The outputs from the Bayesian analysis are detailed in Figure 3 and Table 2. Figure 3A presents the posterior probability density functions of the change-point parameters for the six biomarkers in Sample A. In total, five biomarkers (RF-IgM, ACPA-IgG, RF-IgA, ACPA-IgM, and ACPA-IgA) exhibit pre-diagnosis shifts to the initial trajectories of the RA biomarker concentrations (Table 2). RF-IgM, for instance, exhibits the earliest shift at 8.10 years pre-diagnosis. It is followed by ACPA-IgG (7.43 years pre-diagnosis), RF-IgA (5.39 years), ACPA-IgM (5.27 years), and ACPA-IgA (4.45 years). In contrast, RF-IgG displays a mean change-point around 5.07 years after diagnosis (this is around the latest time post diagnosis that a measurement was taken in Sample A), indicating a post-diagnostic change in its trajectory. None of the 95% credible intervals for magnitude of change include zero (Table 2), indicating that all biomarkers in Sample A show changes in time trend among RA cases.

Figure 3B presents the results of our analysis on Sample B, where the estimated change-points for the eight biomarkers are depicted. Four biomarkers [anti-CCP3 (IgG), anti-citHis1 (IgG), anti-citVim2 (IgG), and anti-citFib (IgG)] showed pre-diagnosis shifts to the initial trajectories of the RA biomarker levels. Anti-CCP3 (IgG) exhibits the earliest change (7.00 years before diagnosis), followed by anti-citHis1 (IgG) (5.70 years), anti-citFib (IgG) (5.44 years), and anti-citVim2 (IgG) (5.11 years). All the biomarkers with pre-diagnosis shifts are IgG variants only (Table 2). IgG biomarkers generally have higher peaks in their posterior density than their IgA counterparts (Figure 3B). Although the posterior changepoint density for the IgG biomarkers has a noticeable peak prior to diagnosis (Figure 3B), the IgA biomarkers have 95% Highest Posterior Density Intervals (HPDI) that extend into post-diagnostic time, indicating uncertainty regarding whether the change-points truly precede diagnosis. The posterior mean correlations among the biomarker-specific change-point estimates are depicted in Figures S1 and S2 of the Supplemental Material. The composite times for anti-citVim2 (IgA) and anti-citFib (IgA) are reported as NA in Table 2, as the posterior probability did not reach 0.9 anywhere within the search window.

Combining change-point timing and magnitude through the composite time $\tau_{k}$ (defined in Methods) yields the ranking in Table 2. In both samples the magnitude information reorders biomarkers. In Sample A, ACPA-IgM ranks ahead of RF-IgA on $\tau_{k}$ despite a slightly later mean change-point, and in Sample B, anti-citVim2 (IgG) ranks ahead of anti-citFib (IgG) on $\tau_{k}$ under the same logic (Table 2).

The censored sensitivity analysis preserved RF-IgM and ACPA-IgG as the two earliest biomarkers in Sample A but ranked RF-IgA ahead of ACPA-IgM, the opposite of the primary truncated model, with RF-IgM and ACPA-IgG retaining the earliest change-points and RF-IgG remaining the sole post-diagnostic biomarker. In Sample B, the four IgG isotypes again had the earliest composite times, with anti-CCP3 (IgG) earliest, so IgG preceded IgA on the composite ranking under both specifications; although anti-citFib (IgA) had the earliest posterior mean change-point, its wide credible interval and small magnitude placed its composite time fifth (Supplement). Under the truncated model the composite times for anti-citVim2 (IgA) and anti-citFib (IgA) were NA, whereas the censored model yielded estimable composite times for all eight Sample B biomarkers. Because the censored model also drops the residual correlation structure in addition to changing how the upper detection limit is handled, the two specifications differ in more than one respect, and their broad agreement should be read as qualitative corroboration rather than a precise test of any single modelling choice.

## Discussion

Our framework characterises within-case acceleration in autoantibody concentrations rather than divergence between cases and controls, and propagates posterior uncertainty in both the timing and magnitude of change. For Sample A, RF-IgM and ACPA-IgG had the earliest mean change-points (8.10 and 7.43 years pre-diagnosis); the composite time reordered ACPA-IgM ahead of RF-IgA relative to the change-point ranking, but the two earliest biomarkers were unchanged. Comparing change-points across isotypes (e.g., IgA versus IgG) may help disentangle the distinct biological processes driving disease evolution at different stages.^27^

Our findings for Sample A provide a different yet complementary perspective to those of Kelmenson and colleagues,^13^ who analysed the same cohort (Sample A). While this previous article identified the earliest time point of statistical divergence between cases and controls, which gives some information as to the development of early RA-related autoimmunity years prior to the identification of arthritis, our model identifies the point of trajectory acceleration within cases, a measure that provides additional information as to underlying pathogenic shifts. For example, we identified the change-point for ACPA-IgG at 7.43 years before diagnosis, suggesting a notable increase in the rate of autoantibody production at this time. This can be reconciled with the earlier divergence reported approximately 17.9 years before diagnosis which may represent the initial, slow-rising phase of autoimmunity that precedes this acceleration.^13^ By modelling the change-point within cases and accounting for inter-biomarker correlation, our approach provides a new layer of information regarding the dynamic evolution of the autoimmune response, whereas the prior analysis focused on establishing the initial point of separation from a healthy state.

In Sample B, only the IgG isotypes of antibodies to citrullinated proteins exhibited pre-diagnostic change-points.^28^ The composite times for anti-citVim2 (IgA) and anti-citFib (IgA) were reported as NA, consistent with the absence of a discernible within-case acceleration for these biomarkers. While mucosal interfaces are hypothesised to be early sites of RA autoimmunity, our results indicate that a systemic IgG response is the consistent and earlier serum signal in this cohort; mucosal processes may nonetheless be occurring but unmeasured in blood. Notable serum IgA responses to these antigens may occur outside the observed window, develop closer to clinical onset, or vary more across individuals. In both samples, some biomarkers shifted only after diagnosis, possibly reflecting post-clinical biology or treatment effects; these patterns warrant further investigation.

Within Sample B, anti-citFib (IgG) had a marginally earlier posterior mean change-point than anti-citVim2 (IgG), but the latter’s substantially larger posterior magnitude placed it ahead under the composite ranking. This illustrates that the combination of timing and magnitude can produce different orderings; the composite metric should be interpreted alongside, not in place of, the underlying δ and γ estimates.

The matched case-control design supports adjustment for confounding by the matching factors. Limitations include reliance on known RA cases, which may limit prospective applicability to populations with abnormal biomarkers but uncertain progression to clinical disease, and a likely true data-generating process with random slopes or higher-order polynomial terms not captured by the current specification. The pair-level random intercept in Sample B further shares a baseline between one case and two controls, which does not fully distinguish between-individual variation among controls within a matched set; a nested individual-level effect could address this but may introduce identifiability challenges given the sparse within-individual observations.

In summary, this Bayesian multivariate segmented framework offers a flexible approach to estimating change-points and their magnitudes in correlated longitudinal biomarkers, with potential application beyond preclinical RA.

## Supplementary Material

Supplement 1

Supplementary data are available online.

## Figures and Tables

**Table 1:** Characteristics of Sample A and Sample B among participants who develop (RA) and their matched counterparts who do not develop (non-RA) rheumatoid arthritis. Variables were measured at enrollment and do not vary over time within participants. Family history of RA was not collected in control (Non-RA) units.

		Sample A (209 pairs; 1:1 match)		Sample B (309 pairs; 1:2 match)	
		Non-RA	RA	Non-RA	RA
**Sex**	Male	108 (51.7%)	108 (51.7%)	306 (49.5%)	153 (49.5%)
	Female	101 (48.3%)	101 (48.3%)	312 (50.5%)	156 (50.5%)
**Race**	White	121 (57.9%)	121 (57.9%)	326 (52.8%)	163 (52.8%)
	Black	55 (26.3%)	55 (26.3%)	158 (25.6%)	79 (25.6%)
	Hispanic/Latino	18 (8.6%)	18 (8.6%)	74 (12.0%)	37 (12.0%)
	Other	15 (7.2%)	15 (7.2%)	60 (9.7%)	30 (9.7%)
**Family history of RA**	Yes	-	52 (24.9%)	-	74 (23.9%)
	No	-	129 (61.7%)	-	189 (61.2%)
	Not Available	209 (100%)	28 (13.4%)	618 (100%)	46 (14.9%)
**Smoking status**	Yes	49 (23.4%)	66 (31.6%)	170 (27.5%)	96 (31.1%)
	No	72 (34.4%)	138 (66.0%)	282 (45.6%)	205 (66.3%)
	Not Available	88 (42.1%)	5 (2.4%)	166 (26.9%)	8 (2.6%)
**Age at diagnosis**	Median (IQR)	36.33 (30.60-42.38)	36.22 (30.81-42.13)	34.85 (28.75-40.63)	34.74 (29.04-40.53)
**Number of serum measures**	N (Average)	734 (3.51)	832 (3.98)	1798 (2.91)	960 (3.11)

**Table 2:** Change-point estimates represent the time (in years before diagnosis) when biomarker trajectories begin to diverge from baseline trend among RA cases. The 95% highest posterior density intervals (HPDI) reflect uncertainty in change-point timing. Composite Time denotes the earliest time point (in years relative to RA diagnosis) at which there is 90% posterior probability that the product of change-point and magnitude exceeds zero among cases. Negative values indicate pre-diagnostic change-points; positive values indicate post-diagnostic change-points. A missing value in the Composite Time column denotes biomarkers for which the posterior probability never reached 0.9 within the search window [−20, 10] years, indicating no reliable evidence of within-case acceleration. This applies to anti-citVim2 (IgA) and anti-citFib (IgA) in Sample B. **Abbreviations**: RF=rheumatoid factor; Ig=immunoglobulin; ACPA=anti-citrullinated protein antibody; CCP3=cyclic citrullinated peptide-3; cit=citrullinated; His=histone; Vim=vimentin; Fib=fibrinogen; HPDI=highest posterior density interval.

BIOMARKER	COMPOSITE TIME (YEARS)	AVERAGE CHANGE- POINT [95% HPDI]	MAGNITUDE OF CHANGE MEAN [95% HPDI]
SAMPLE A			
RF IgM	−6.58	−8.10 [−10.47, −5.73]	3.53 [2.58, 4.52]
ACPA IgG	−6.41	−7.43 [−9.33, −5.76]	12.35 [9.36, 15.15]
ACPA IgM	−4.04	−5.27 [−7.62, −3.34]	7.11 [4.10, 9.56]
RF IgA	−3.90	−5.39 [−8.89, −2.83]	2.28 [1.40, 3.12]
ACPA IgA	−3.35	−4.45 [−7.90, −2.60]	8.89 [5.31, 11.43]
RF IgG	5.50	5.07 [4.13, 5.57]	418.67 [27.84, 1285.80]
SAMPLE B			
anti-CCP3 (IgG)	−5.96	−7.00 [−8.48, −5.29]	10.88 [8.90, 13.09]
anti-citHis1 (IgG)	−4.29	−5.70 [−8.13, −3.42]	1.02 [0.70, 1.38]
anti-citVim2 (IgG)	−4.09	−5.11 [−6.74, −3.52]	9.20 [6.93, 11.68]
anti-citFib (IgG)	−3.94	−5.44 [−7.54, −2.99]	3.21 [2.14, 4.27]
anti-CCP3 (IgA)	9.50	3.35 [−7.99, 10.00]	0.39 [−0.05, 1.18]
anti-citHis1 (IgA)	9.61	1.22 [−7.18, 10.00]	0.56 [−0.56, 2.26]
anti-citVim2 (IgA)	-	−1.01 [−12.03, 10.00]	0.07 [−1.93, 1.99]
anti-citFib (IgA)	-	−1.79 [−10.44, 10.00]	0.10 [−0.72, 0.89]

## Data Availability

Because of regulatory issues, the data from this study are not available. However, code to implement this methodology can be found here: https://github.com/yonwol3/ra-biomarker

## Key Messages

- We fit a Bayesian multivariate segmented regression to jointly estimate unknown change-points and magnitudes of change across correlated autoantibody trajectories in preclinical rheumatoid arthritis.
- RF-IgM and ACPA-IgG exhibited the earliest pre-diagnostic trajectory acceleration (approximately eight and 7.4 years before diagnosis, respectively), and only IgG isotypes showed consistent pre-diagnostic shifts in a novel panel of anti-citrullinated protein antibodies.
- A composite metric integrating both the timing and magnitude of autoantibody change provides a different ranking of biomarkers than change-point estimation alone, which may inform future screening and prevention strategies.
