# Supplementary material for: Detecting change-points in preclinical rheumatoid arthritis biomarkers using Bayesian multivariate segmented regression
Source: medRxiv. 2026 May 25:2026.05.22.26353892. Preprint. [Version 1] doi: 10.64898/2026.05.22.26353892 (PMC13232369; doi:10.64898/2026.05.22.26353892)
Supplement: Supplementary file 1 [file media-1.pdf]

# Supplement to "Detecting change-points in preclinical rheumatoid arthritis biomarkers using Bayesian multivariate segmented regression"

Yonatan F. Wolde, Alexandria M. Jensen, Brandie D. Wagner, Jess D. Edison, Marie L. Feser, Michael Mahler, Kevin D. Deane, and Kevin P. Josey

This supplement provides additional details on the prior specification for the primary truncated model, and reports a sensitivity analysis that treats values at the upper detection limit as right-censored rather than truncated.

**Truncated Model Prior Distribution**

We suppose the regression parameters $\boldsymbol{\beta}_1$, $\boldsymbol{\beta}_2$, $\boldsymbol{\gamma}$ and $\boldsymbol{\theta}$ have a multivariate normal distribution, with mean zero and a diagonal covariance matrix with diagonal elements equal to $10^6$. For $k = 1,2, \dots, K$, the variance components $\sigma_k$ and $\omega_k$ both have a half-Cauchy(0, 2) prior.[1] Change-points $\delta_k$ have a continuous uniform over $[-20, 10]$ years.[2] The correlation matrix (in the truncated model) $\boldsymbol{\Phi}$ uses an LKJ(1) prior.[3] All choices are non- or weakly informative.

**Right Censored Sensitivity Analysis**

We conducted a sensitivity analysis on Sample A and Sample B which assumes censoring instead of truncation at the upper level of detection. Unlike the model in the main manuscript, this model does not explicitly account for correlation between exposures with a prior on the covariance matrix. For a participant $i = 1,2, \dots, n$, with measurement $j = 1,2, \dots, m_i$, and for biomarker $k = 1, 2, \dots, K$ ($K = 6$ for Sample A; $K = 8$ for Sample B), we denote the outcome vector as $\boldsymbol{Y}_{ij} = \left[Y_{ij1}, Y_{ij2}, \dots, Y_{ijK}\right]^T$ with each element $Y_{ijk}$ constrained to lie between a lower bound of 0. That is, the response distribution follows

$$\boldsymbol{Y}_{\text{ij}} \sim \text{Truncated Normal}\left(\boldsymbol{\mu}_{ij}, \boldsymbol{\Sigma}, \boldsymbol{L} = \boldsymbol{0}_K\right).$$

If a biomarker concentration $Y_{ijk}$ equals its maximum detectable value, $U_k$, the value is treated as right-censored, with its likelihood contribution marginalised analytically using the upper-tail

normal probability.[4,5] Another modification necessary to run the model with Stan is in the correlation specification for the biomarker outcomes. We were unable to explicitly model $\boldsymbol{\Sigma}$ with a single prior. Instead, we define $\boldsymbol{\Sigma} = \text{diag}(\boldsymbol{\sigma}^2)$, i.e. without modelling the correlation matrix $\boldsymbol{\Phi}$ as in our main truncated model. The matrix $\text{diag}(\boldsymbol{\sigma}^2)$ is the diagonal matrix of the squared residual standard deviations $\boldsymbol{\tau} = (\tau_1, \tau_2, \ldots, \tau_K)$. The random intercept $\boldsymbol{\alpha}_{l[i]} \sim N(\boldsymbol{\theta}, \boldsymbol{\Omega})$ is defined just as it is in the main truncated model, and the mean model remains unchanged.

The regression parameters $\boldsymbol{\beta}_1, \boldsymbol{\beta}_2, \boldsymbol{\gamma}$, and $\boldsymbol{\theta}$ were each assigned a multivariate normal prior with mean zero and a diagonal covariance matrix with a larger variance of $10^8$. The hyperparameters $\sigma_k$ and $\omega_k$ were assigned a weakly informative half-Cauchy(0, 2) prior distributions. The change point for each biomarker $\delta_k$ was assigned a uniform non-informative prior, with equally plausible values from 20 years before diagnosis to 10 years after diagnosis. Unlike the primary truncated model, which employs an LKJ prior on the correlation matrix $\boldsymbol{\Phi}$, computational constraints in Stan's handling of censored multivariate likelihoods necessitated an independence assumption for the residual covariance structure in this sensitivity analysis. The two models therefore differ in two respects (handling of values above the upper detection limit and residual correlation structure), so agreement between their posterior summaries should not be interpreted as a narrow test of any one of these choices. The results of this model are summarised in Table S1, where biomarkers are ranked by the earliest composite time measure (defined in the main model).

**Sensitivity Analysis Results**

For Sample A, the censored model preserves the two earliest biomarkers (RF IgM and ACPA IgG) but ranks RF IgA earlier than ACPA IgM (-5.35 vs -4.91 years), the opposite of the primary truncated model in which ACPA IgM (-4.04 years) precedes RF IgA (-3.90 years). In addition, apart from RF IgG, all biomarkers are estimated to have a pre-diagnosis change-point, while RF IgG is still estimated to have a change point after diagnosis (Table S1). Figure S3 shows the change-point posterior densities for Sample A biomarkers, while Figure S4 shows the change-point posterior densities for Sample B biomarkers. The posterior densities of Sample A biomarkers for the most part resemble those of our main truncated model.

For Sample B, all the biomarkers had a change-point before diagnosis except for anti-citVim2(IgA) (Table S1). Notably, anti-CCP3 (IgG) has the earliest Composite Time even though anti-citFib (IgA) had the earliest estimated change-point, but anti-citFib (IgA) was ranked fifth once incorporating magnitude of change information, underscoring the importance of considering the composite measure (change point + magnitude of change) instead of just using the change-point parameter (Table S1).  There is much overlap in Sample B biomarkers' density (Figure S4), but some biomarkers (anti-CCP3 (IgG & IgA), anti-citVim2 (IgG), anti-citHis1(IgG) and anti-citFib (IgG)) have clearly noticeable peaks, while others have flatter densities, with a wide range of 95% HPDI for the change-point estimates (Table S1). Overall, IgG isotypes change earlier than their IgA counterparts.

| Sample A | | | |
| --- | --- | --- | --- |
| **Biomarker** | **Composite Time (Years)** | **Average Change-point [95% HPDI]** | **Magnitude of Change Mean [95% HPDI]** |
| RF IgM | -7.68 | -9.41 [-12.46, -6.62] | 4.18 [3.14, 5.28] |
| ACPA IgG | -6.97 | -8.11 [-10.02, -6.39] | 14.32 [11.45, 17.20] |
| RF IgA | -5.35 | -7.97 [-11.91, -4.32] | 2.27 [1.41, 3.12] |
| ACPA IgM | -4.91 | -6.55 [-9.15, -4.19] | 7.96 [5.42, 10.50] |
| ACPA IgA | -4.36 | -5.89 [-8.48, -3.78] | 8.12 [5.79, 10.69] |
| RF IgG | 5.34 | 4.85 [4.31, 5.46] | 898.76 [62.16, 2004.84] |
| **Sample B** | | | |
| anti-CCP3 (IgG) | -6.18 | -7.04 [-8.37, -5.70] | 12.17 [10.24, 14.17] |
| anti-citHis1 (IgG) | -4.73 | -5.79 [-7.60, -3.98] | 1.08 [0.81, 1.36] |
| anti-citVim2 (IgG) | -4.67 | -5.55 [-7.13, -4.31] | 9.15 [7.25, 11.08] |
| anti-citFib (IgG) | -4.53 | -5.45 [-7.15, -4.04] | 3.49 [2.61, 4.30] |
| anti-citFib (IgA) | -4.37 | -8.46 [-17.48, -2.50] | 0.16 [0.06, 0.28] |
| anti-CCP3 (IgA) | -2.41 | -7.56 [-19.86, -1.39] | 0.11 [0.02, 0.23] |
| anti-citHis1 (IgA) | -2.15 | -5.50 [-14.55, 1.08] | 0.29 [0.08, 0.52] |
| anti-citVim2 (IgA) | 3.15 | -6.69 [-19.98, 7.40] | 0.37 [-0.24, 2.08] |

**Table S1:** Summaries of posterior censored model output for sample A and sample B resembling Table 2. **Abbreviations**: RF=rheumatoid factor; Ig=immunoglobulin; ACPA=anti-citrullinated protein antibody; CCP3=cyclic citrullinated peptide-3; cit=citrullinated; His=histone; Vim=vimentin; Fib=fibrinogen; HPDI=highest posterior density interval.

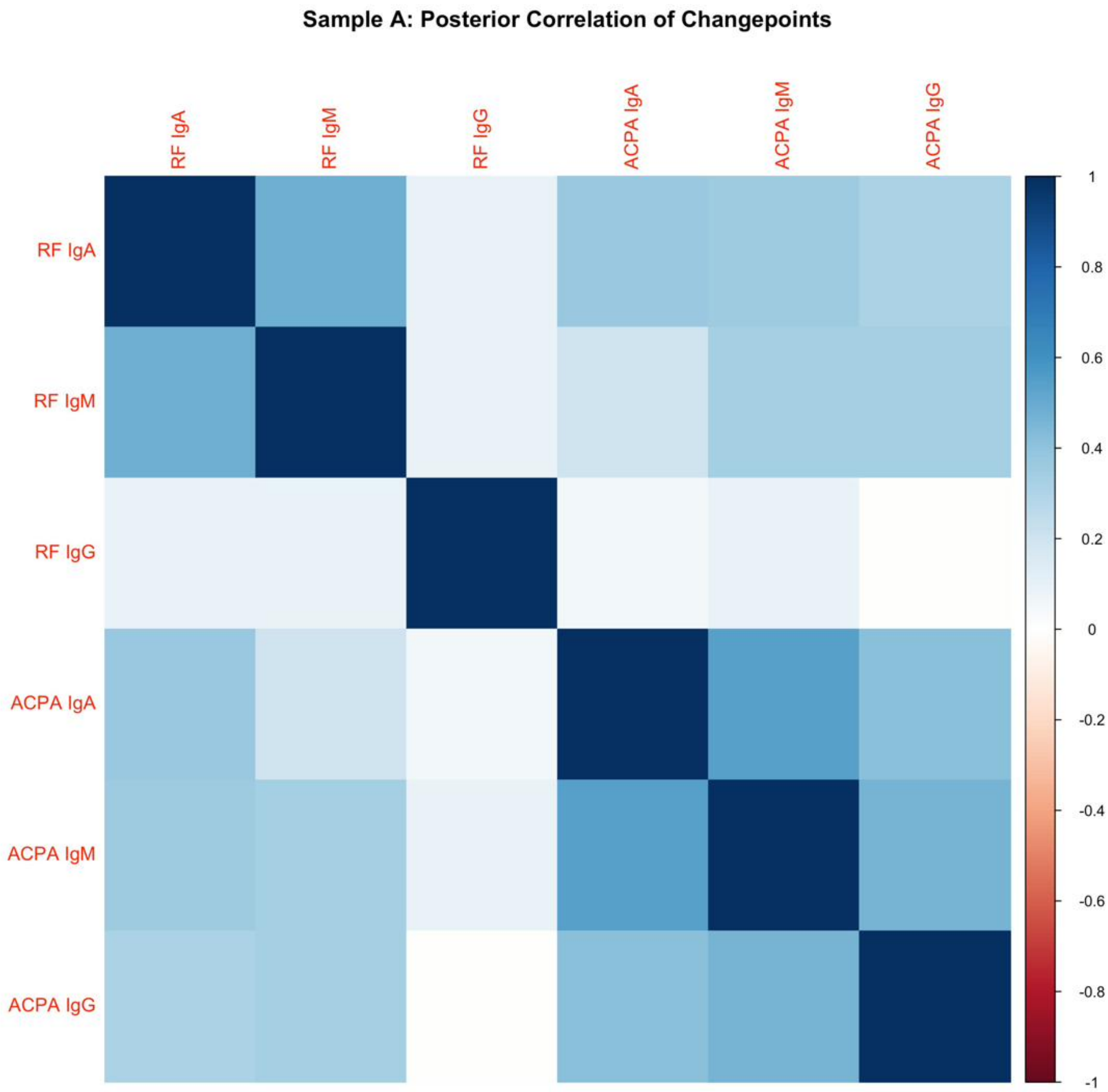


**Figure S1.** Posterior mean correlation matrix of the change-point estimates for the six biomarkers in Sample A under the truncated normal model specification. Each cell displays the posterior mean of the pairwise correlation between biomarker-specific change-point parameters across MCMC draws, with colour intensity indicating the strength and direction of correlation.

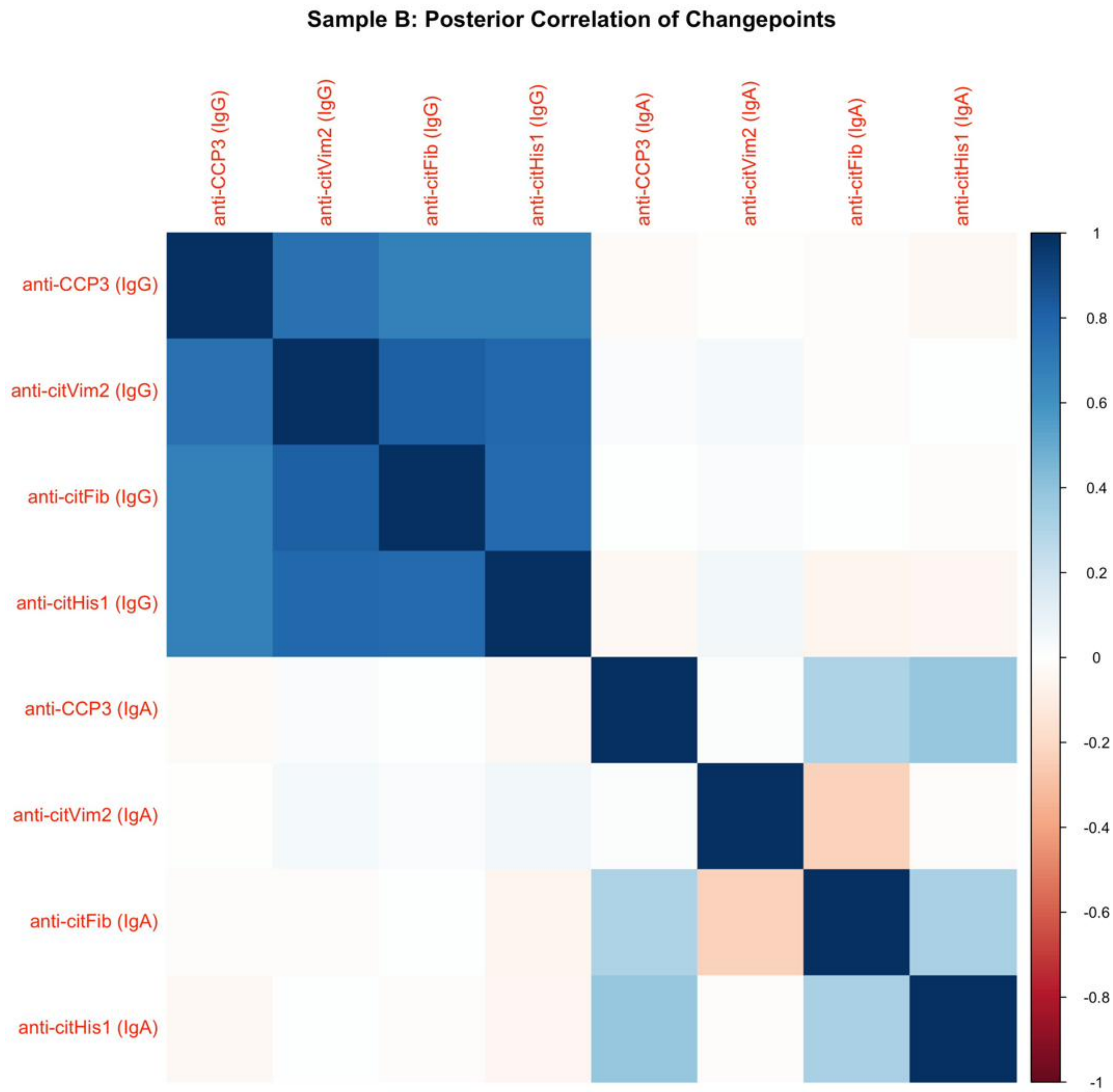


**Figure S2.** Posterior mean correlation matrix of the change-point estimates for the eight biomarkers in Sample B under the truncated normal model specification. Each cell displays the posterior mean of the pairwise correlation between biomarker-specific change-point parameters across MCMC draws, with colour intensity indicating the strength and direction of correlation.

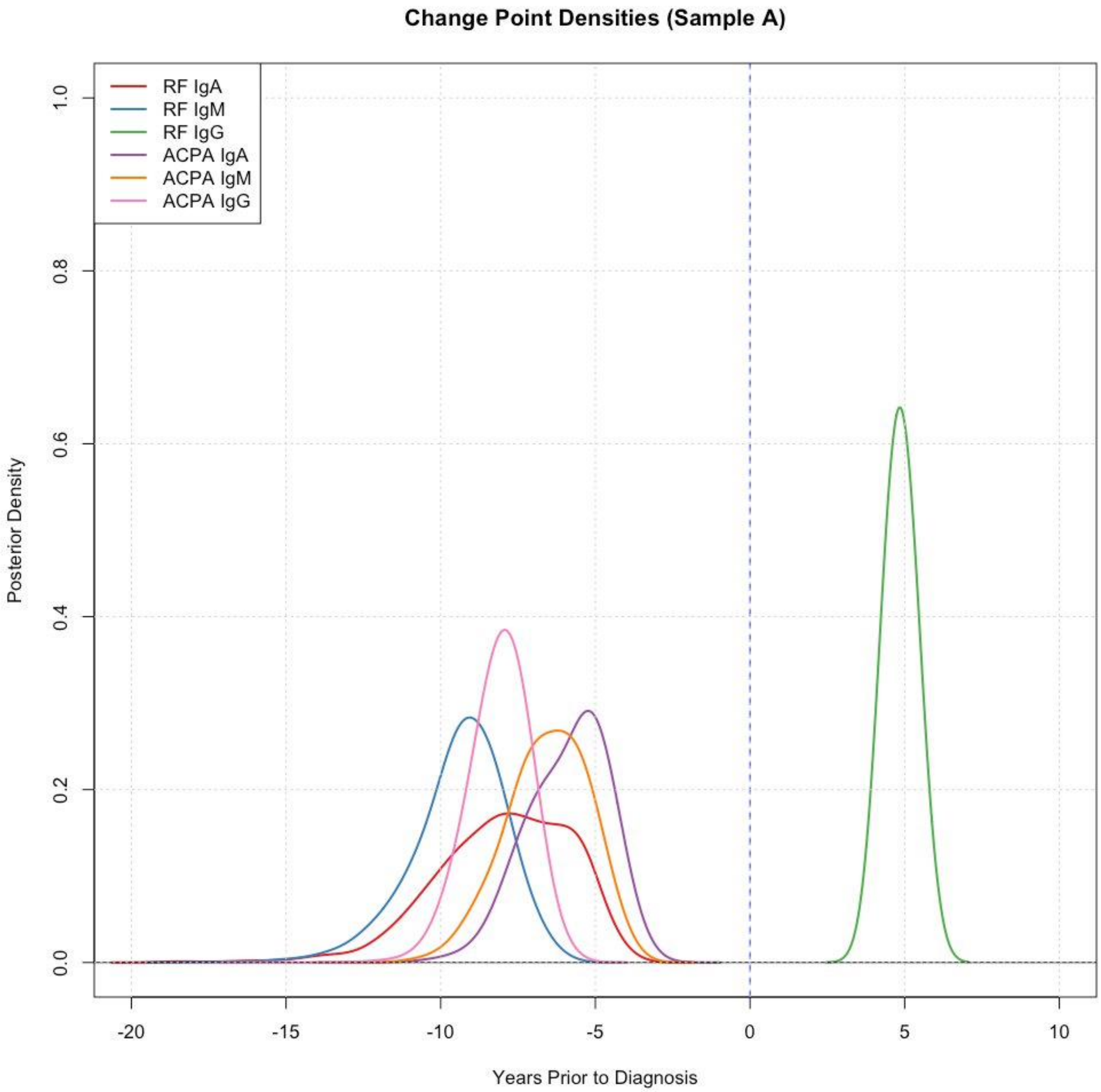


**Figure S3.** Posterior probability density functions of the change-point parameter for each biomarker from the censoring sensitivity model. It depicts posterior change point density for the six biomarkers in Sample A (labels in the top-left corner). The peak of each density plot corresponds to the most probable change-point estimate.

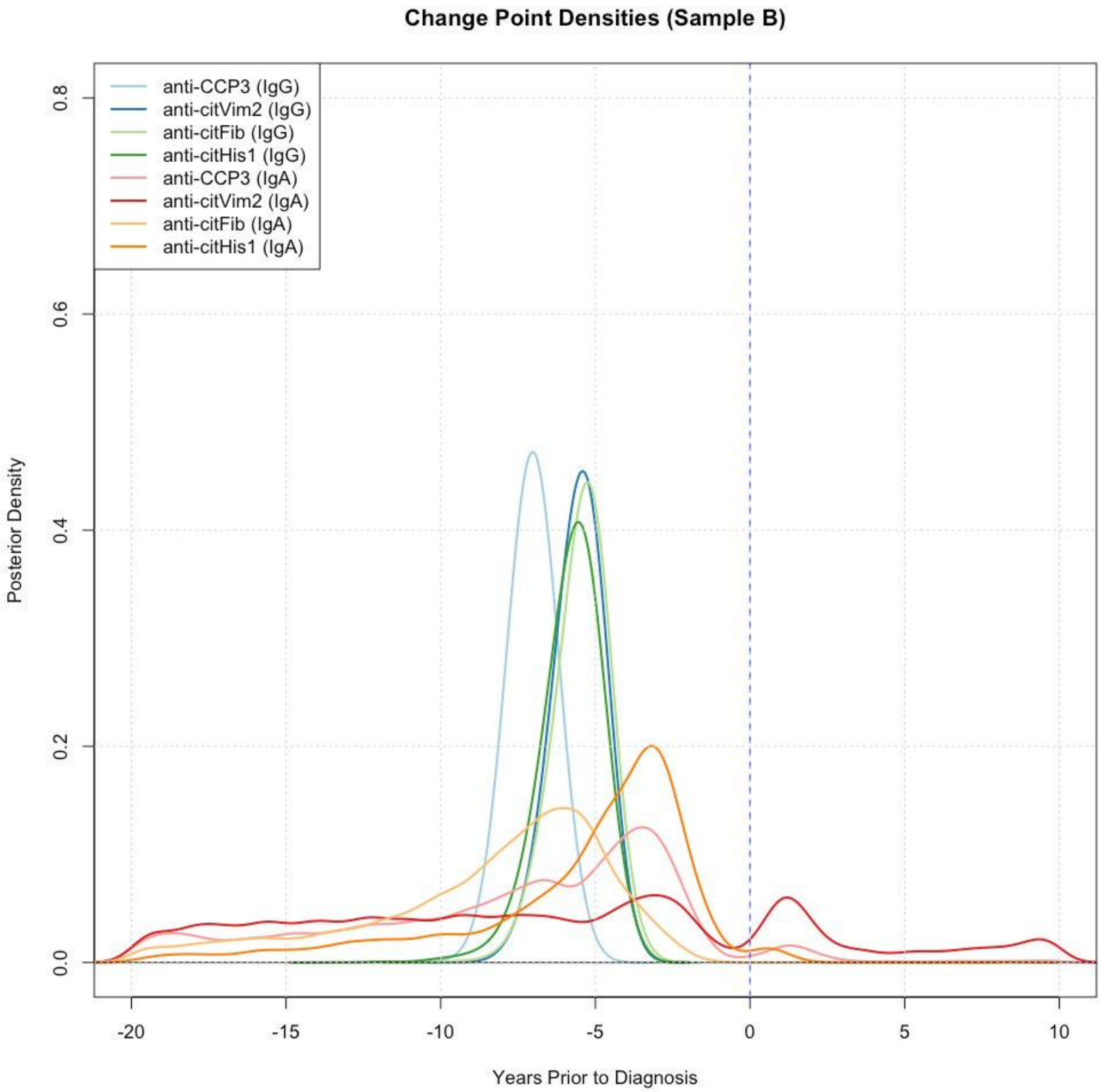


**Figure S4.** Posterior probability density functions of the change-point parameter for each biomarker from the censoring sensitivity model. It depicts posterior change point density for the eight biomarkers in Sample B (labels in the top-left corner). The peak of each density plot corresponds to the most probable change-point estimate.

## References


1. Gelman A. Prior distributions for variance parameters in hierarchical models (comment on article by Browne and Draper). *Bayesian Anal*. 2006;1(3):515-534. doi:10.1214/06-BA117A
2. Gössl C, Küchenhoff H. Bayesian analysis of logistic regression with an unknown change point and covariate measurement error. *Stat Med*. 2001;20(20):3109-3121. doi:10.1002/sim.928
3. Lemoine NP. Moving beyond noninformative priors: why and how to choose weakly informative priors in Bayesian analyses. *Oikos*. 2019;128(7):912-928. doi:10.1111/oik.05985
4. Chen H, Quandt SA, Grzywacz JG, Arcury TA. A Bayesian multiple imputation method for handling longitudinal pesticide data with values below the limit of detection. *Environmetrics*. 2013;24(2):132-142. doi:10.1002/env.2193
5. van Dyk DA, Meng X-L. The art of data augmentation. *J Comput Graph Stat*. 2001;10(1):1-50. doi:10.1198/10618600152418584
